# Pseudo-hyperacute T-waves in a giant thoracic aortic aneurysm

**DOI:** 10.1093/ehjcr/ytae502

**Published:** 2024-09-14

**Authors:** Masahiro Ishikura, Yoshiaki Kawase, Hirooki Higami, Hitoshi Matsuo

**Affiliations:** Department of Cardiology, Gifu Heart Center, 4-14-4 Yabutaminami, Gifu 500-8384, Japan; Department of Cardiology, Gifu Heart Center, 4-14-4 Yabutaminami, Gifu 500-8384, Japan; Department of Cardiology, Gifu Heart Center, 4-14-4 Yabutaminami, Gifu 500-8384, Japan; Department of Cardiology, Gifu Heart Center, 4-14-4 Yabutaminami, Gifu 500-8384, Japan

## Summary

An 83-year-old man presented to our hospital with dyspnoea on exertion. An electrocardiogram (ECG) showed hyperacute T-waves, suggesting acute coronary syndrome (ACS). However, transthoracic echocardiography revealed that the heart was being compressed by a giant thoracic aortic aneurysm (TAA). A computed tomography (CT) scan also showed the TAA, which was 11 cm in a diameter, with no evidence of rupture. After urgent thoracic endovascular aortic repair (TEVAR), the patient’s subjective symptoms improved and the ECG changes present on admission also improved. The present case illustrates that compression of the heart by a giant TAA induces hyperacute T-waves mimicking ACS.

## Case description

**Figure 1:**
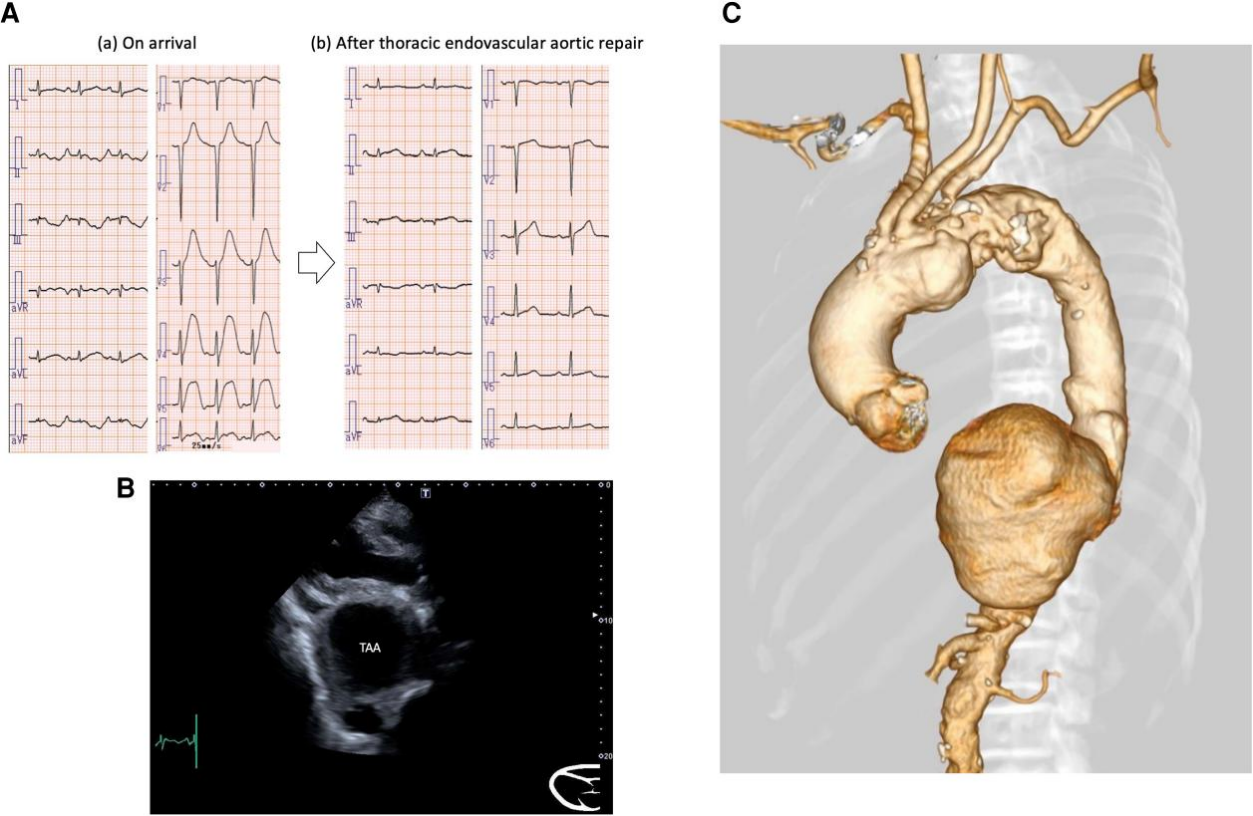
(*A*) Initial electrocardiogram on arrival (a): hyperacute T-waves in V2∼5 and ST depression in Ⅱ, Ⅲ, and aVF leads. Electrocardiogram after thoracic endovascular aortic repair (b): hyperacute T-waves in V2∼5 leads were normalized. (*B*) Echocardiogram in parasternal long-axis revealed the heart was being compressed by a giant thoracic aortic aneurysm. (*C*) The computed tomography scan showed a giant thoracic aortic aneurysm.

An 83-year-old man presented to our hospital with dyspnoea on exertion. His blood pressure was 156/109 mmHg. An ECG showed a heart rate of 116 b.p.m., hyperacute T-waves in V2∼5 and ST depression in Ⅱ, Ⅲ, and aVF leads (*Figure 1A*). We first suspected an ACS. However, transthoracic echocardiography revealed that the heart was being compressed by a giant TAA (*Figure 1B*, Supplementary material online, *Video S1*). A CT scan also showed the TAA, which was 11 cm in a diameter, with no evidence of rupture (*Figure 1C*). Laboratory tests showed only slightly elevated high-sensitivity cardiac troponin T (0.034 ng/mL) and no hyperkalaemia. The TAA was so large that the risk of rupture was considered high, and urgent TEVAR was performed. After TEVAR, the patient’s subjective symptoms improved and the ECG changes present on admission also improved (*Figure 1A*). Absence of severe coronary artery stenosis was confirmed with cardiac CT during hospitalization. The patient was discharged on Day 12.

Ono *et al*.^1^ have reported a similar case of pseudo-hyperacute T-waves. In their case, a heart was compressed by a giant hiatal hernia. In common with our case, mechanical external compression of the heart caused pseudo-hyperacute T-waves. There have been also some case reports of posterior mediastinal haematoma with cardiac compression associated with TAA rupture.^2^ Hoshika *et al.* have reported a case of acute myocardial infarction due to right coronary artery compression by mediastinal haematoma associated with TAA rupture. In their case, the ECG showed ST-segment elevation. However, to the best of our knowledge, there have been no reported cases of ECG abnormalities due to cardiac compression caused by unruptured TAAs. In the case of a hyperacute T-wave in ECG, other possibilities than ACS should be considered.

## Supplementary Material

ytae502_Supplementary_Data

## Data Availability

The data underlying this article will be shared on reasonable request to the corresponding author.
